# An Exploration of the Cellular Microenvironment of the Female Pig Urethra: Translational Insights for Urological Research

**DOI:** 10.3390/biology14010031

**Published:** 2025-01-03

**Authors:** Agustín Cartes, Caroll Stoore, María Soledad Baquedano, Christian Hidalgo, Felipe Lillo, Eduardo Landerer, Galia Ramírez-Toloza, Rodolfo Paredes

**Affiliations:** 1 Escuela de Medicina Veterinaria, Facultad de Ciencias de la Vida, Universidad Andres Bello, República 239 3° Piso, Santiago 8370146, Chile; agustin.cartes@unab.cl (A.C.); cstoorep@gmail.com (C.S.); s.baquedanoj@gmail.com (M.S.B.); felipe.lillo@unab.cl (F.L.); 2 Programa de Doctorado en Ciencias Silvoagropecuarias y Veterinarias, Campus Sur, Universidad de Chile, Santa Rosa 11315, La Pintana, Santiago 8820808, Chile; 3 Núcleo de Investigación en One Health (NIOH), Facultad de Medicina Veterinaria y Agronomía, Universidad de las Américas, Santiago 8370065, Chile; chidalgo@udla.cl; 4 Unidad Urología, Centro de Cirugía Robótica, Clínica Indisa, Santa María 1810, Providencia, Santiago 7520440, Chile; elanderer@gmail.com; 5 Departamento de Medicina Preventiva Animal, Facultad de Ciencias Veterinarias y Pecuarias, Universidad de Chile, Santa Rosa 11735, La Pintana, Santiago 8820808, Chile; galiaram@uchile.cl

**Keywords:** pig urethra, urethral anatomy, urological model, translational research

## Abstract

Urinary incontinence is a widespread issue, particularly among women, with effective treatments remaining elusive. The pig, and especially the female pig, stands as a promising animal model for the study of this condition, due to its anatomical similarities to humans. The aim of this study was to explore the largely uncharted muscular structure of the female pig urethra, linking urethral muscle dysfunction to incontinence. We examined histological sections from the urethras of six sows using Hematoxylin-Eosin and Masson’s trichrome staining for morphometric analysis. The statistical significance of cellular disposition was determined through analysis of variance (ANOVA), followed by a Tukey post hoc test to elucidate specific inter-group differences. Our analysis revealed segment-specific epithelial differences, including variations in cell layers, sparse acinar glands, rich vasculature, and distinct muscle fibers with diverse regional distributions. Notably, significant differences in muscular area and tissue distribution were identified between the proximal, middle, and distal segments of the urethra (*p* < 0.001). The observed anatomical variations, along with the cellular similarities between pigs and humans, establish the female pig as a crucial translational model for advancing urological research. Specifically, these findings provide a foundation for the development of innovative therapeutic strategies and surgical techniques that can be directly applied to improve outcomes in human urological conditions.

## Simple Summary

Urinary incontinence is a common and significant condition, particularly among women, yet effective treatments remain limited due to gaps in our understanding of its underlying mechanisms. This study explores the anatomy of the female pig urethra, positioning it as a promising model for urological research due to its anatomical similarities to humans. By analyzing histological sections of urethras from six sows, we identified significant structural variations in muscle distribution and tissue organization across different urethral segments. These findings included distinct epithelial configurations, sparse glands, and variations in muscular and luminal areas. Urodynamic studies further revealed differences in pressure profiles, enhancing our understanding of urethral function. The observed anatomical parallels between pig and human urethras underscore the translational potential of this model for advancing research in urinary incontinence. By providing new insights into urethral anatomy and function, this study lays the groundwork for developing innovative therapies and surgical techniques aimed at improving the quality of life for individuals affected by this condition.

## 1. Introduction

Urinary incontinence (UI) is a widespread condition affecting millions globally, with women exhibiting a disproportionately higher prevalence compared to men. Epidemiological studies estimate the prevalence at 13.1% among women and 5.4% among men [1]. Despite its profound impact on quality of life, the mechanisms underlying UI are not fully understood, presenting significant barriers to the development of effective therapeutic strategies [2]. Both pharmacological and non-pharmacological interventions have demonstrated efficacy in improving UI outcomes compared to no treatment. Behavioral therapy, either as a standalone intervention or combined with other treatment modalities, has been found to outperform pharmacological therapies alone in managing stress and urgency UI [3].

Among injectable therapies, urethral bulking agents have emerged as promising options. Bulkamid^®^ has shown short-term efficacy rates ranging from 30% to 90% and long-term efficacy of 42% to 70%, while Macroplastique^®^ has demonstrated short-term efficacy of 40% to 85% and long-term efficacy ranging from 21% to 80% [4]. These agents offer viable solutions for managing UI; however, the variability in their long-term efficacy highlights the importance of tailoring treatment strategies to meet individual patient needs and optimize outcomes. In this context, animal models have become crucial tools to study the pathophysiology of urinary incontinence and to develop new therapies. Among animal models, female pigs have emerged as a promising option for studying urinary incontinence due to their anatomical and physiological similarities to humans [5]. However, despite their potential, there is still a lack of knowledge regarding the anatomy of the pig’s urethra and its role as a model in understanding the pathogenesis of urinary incontinence. Few studies have examined the anatomy and contractile properties of the porcine urinary tract, specifically the bladder neck and proximal urethra in sows [6,7,8]. However, these investigations do not provide an in-depth analysis of the entire urethra or the cellular microenvironment that shapes the urothelium’s composition. Meanwhile, other efforts have primarily focused on defining the significance of the urethral sphincter in the male pig’s urethra [5].

The urethra is a crucial structure responsible for eliminating urine from the body in both male and female mammals. The muscular component of the urethra plays a crucial role in facilitating proper urine flow and preventing urinary tract disorders. In sows, previous research has shown that the urethra is more complex compared to other domestic species such as cats, dogs, or mice [6]. It exhibits a distinct muscular architecture, with multiple layers of striated and smooth muscle fibers. Notably, the smooth muscle fibers are arranged in a helical pattern, which may influence urine flow dynamics [7]. Additionally, recent research focusing on the dynamic properties of urethras has compared the mechanical behavior of the sow’s urethra to that of the human urethra, establishing it as a model for studying the human urinary system [9]. Specifically, porcine urethras are considered a suitable substitute for investigating human urethral mechanics due to their similar dimensions, anatomical features, and tissue properties [10]. Nonetheless, the characterization of the cellular anatomy of the female pig urethra remains poorly understood.

Recent studies in women have shed some light on the role of the urethra in urinary continence, with evidence suggesting that urethral muscular dysfunction is a crucial factor in the development of incontinence [11]. Additionally, new techniques, such as pelvic floor ultrasound and urodynamic studies, have provided insight into the biomechanical properties of the urethra and its relationship with incontinence [12,13].

In this context, pig urodynamic studies have emerged as a valuable tool for comprehensively understanding various urological pathologies. These studies leverage the anatomical and physiological similarities between porcine and human urinary systems, making pigs a potential experimental model. The use of pigs in urodynamic research offers several advantages, such as their larger size compared to rodents, which allows for more accurate instrumentation and data collection. Moreover, the availability of advanced imaging techniques and minimally invasive surgical procedures in pig models enables closely mimicking human urological conditions, leading to enhanced translational relevance [14].

The anatomical and physiological similarities between female pigs and humans, particularly in the urethra, suggest that pigs may serve as effective models for studying human urological pathologies. Female pigs have demonstrated promise as models for stress urinary incontinence due to their structural urethral characteristics and responsiveness to interventions that mimic human conditions, such as urethral sphincter deficiency [15]. Preliminary data indicate that the urethral anatomy and function in pigs closely replicate the sphincteric dynamics observed in humans, making them particularly suitable for investigating the mechanisms of continence and the development of therapeutic interventions [16,17,18].

Furthermore, pigs can provide valuable information about infectious urinary diseases, given their physiological similarity to humans. Experimental models involving uropathogenic *Escherichia coli* have demonstrated both acute and chronic pathogenic mechanisms and host immune responses relevant to urinary tract infections (UTIs). These models offer a foundation for studying ascending and invasive infections, contributing to the understanding of human UTIs [19]. However, limitations exist in using pigs for modeling certain pathologies, such as tumoral diseases, as spontaneous urethral tumors are rare in pigs and their tumor biology differs from that of humans [20,21].

It is important to acknowledge that urinary incontinence poses a considerable challenge for healthcare providers and researchers. In the United States alone, over $12 billion USD are spent annually in the management of this condition [22]. Moreover, animal models, particularly the female pig, present promising avenues for delving into the underlying mechanisms of this condition. As such, the primary objective of this investigation is to provide a comprehensive macroscopic and microscopic characterization of the female pig urethra. Specifically, the study aims to delineate the structural organization of the urethral musculature, identify key anatomical features relevant to its function, and establish its translational relevance for advancing research in urological pathologies such as urinary incontinence.

## 2. Materials and Methods

### 2.1. Ethics

All procedures were carried out and obtained certification by the Bioethics Committee of Andres Bello University according to act No. 027-2020, and received authorization from the Regional Health Ministerial Secretariats (SEREMI) to obtain the samples.

### 2.2. Animals

Urethrovesical units were obtained at a local slaughterhouse from six adult female pigs, weighing between 100–110 kg, immediately after death.

A sow was employed for profilometric assessment and subsequently utilized in a clinical surgical investigation conducted by our research team. The sow received standard care for 24 h, with ad libitum access to food and water. It was housed in a controlled, tranquil environment with appropriate lighting and temperature management, aimed at minimizing environmental stressors.

### 2.3. Tissue Preparation

Urethras from six pigs were transversal and longitudinally sectioned to macroscopically characterize their differences, and measure variations in the total, muscular, and luminal thickness. The transverse portion of the urethra was divided into four segments, each approximately 1 cm in length, and labeled as proximal (Pu), mid-proximal (MPu), mid-distal (MDu), and distal (Du) in the cranio-caudal direction, following a previously described protocol [23]. All sections were included in the microscopic analysis. These segments were selected based on their anatomical significance and the limited knowledge regarding their urothelial composition and the functional intraurethral pressure specific to each segment.

### 2.4. Histology and Morphometry

After routine preparation of paraffin-embedded tissues for histology for each part of the total urethra, four transversal sections were chosen at approximately 5 mm intervals, stained with Hematoxylin-eosin and Masson trichrome staining. The histological sections were analyzed with an Olympus CKX41 microscope (Olympus, Tokyo, Japan) equipped with a BASLER acA2040-55uc USB 3.0 camera digital scanner with a Sony IMX265 CMOS sensor (BASLER, Schleswig-Holstein, Germany) and a manual WSI image analysis software. Morphometric analysis was performed on Masson trichrome-stained sections. For each transversal section, four tissues were quantified: circular smooth muscle (CSm), longitudinal smooth muscle (LSm), lamina propria (LP), and lumen (L). Each slide was scanned, and its minor diameter was manually traced, whose length was automatically measured by the software ImageJ version 1.44p (https://imagej.net/ij/). The determination of the tissue distribution involved a color-based segmentation approach, whereby distinctions were made among tissue types within the specimen. This process encompassed the differentiation between interstitial tissue, constituting the extracellular matrix, and myofibers, which denote muscle tissue. The quantification of tissue content was achieved through a meticulous assessment of their respective spatial extents. To elucidate the relative composition of these tissue types, the proportion of muscular tissue to the total tissue content was derived by dividing the area occupied by muscular tissue by the overall area of the specimen.

### 2.5. Urodynamic Studies Protocol

A healthy adult sow was selected for the study. Prior to the procedures, the subject underwent an 8-h fasting period for solid food and a 4-h fasting period for liquids, adhering to standard protocols to minimize the risk of aspiration. A comprehensive clinical examination was subsequently conducted to confirm the suitability of the subject for the study.

To initiate sedation, a premedication regimen was administered intramuscularly, comprising Tiletamine + Zolazepam (Zoletil^®^) at 2 mg/kg, Dexmedetomidine at 0.01 mg/kg, and Tramadol at 5 mg/kg. This combination was selected for its synergistic properties, effectively providing sedation and analgesia while minimizing stress and adverse hemodynamic effects. Once a sedated state was achieved, the sow was promptly transferred to a preparation room within 3 min, where preoxygenation was administered using a veterinary-specific face mask. Additionally, two intravenous lines were established to facilitate the administration of fluids and intravenous medications as required.

Anesthesia was induced intravenously using Propofol at a dose of 3 to 5 mg/kg, allowing for a rapid and smooth induction process. Orotracheal intubation was subsequently performed, and the sow was connected to an inhalation anesthesia machine, with the initial fraction of inspired oxygen (FiO_2_) set at 0.6. Isoflurane was selected as the inhalation agent due to its predictable anesthetic depth and rapid recovery profile.

The multimodal anesthesia protocol employed a combination of inhalation anesthesia with a continuous infusion of Ketamine (1 mg/kg/h) and Dexmedetomidine (0.001 mg/kg). This approach was designed to achieve balanced anesthesia, minimizing potential complications associated with anesthesia and maintaining physiological stability. Given the sensitivity of urethral pressure measurements to sedation and anesthesia, the protocol was specifically tailored to preserve urethral tone and function, ensuring the reliability and accuracy of the data collected.

With the sow in a lateral position, a pressure transducer was carefully inserted into the urethra through cystoscopy. The transducer was equipped with pressure sensors distributed along its length to obtain a comprehensive urethral pressure profile. The pressure data were recorded as the probe was withdrawn from the urethra, maintaining a constant bladder volume of 4 mL/kg. Multiple measurements were taken at different time points to capture variations in pressure profiles.

Following the intervention, the sow underwent anesthesia recovery period, and then was subsequently relocated to the laboratory animal center in a healthy status.

### 2.6. Statistical Analysis

A meticulous examination of the histomorphometric data for each distinct urethral segment included the calculation of essential statistical parameters, such as mean, standard deviation, mode, and medians. To determine the statistical significance, regions with a probability of less than 5% (*p* = 0.05) were rigorously scrutinized. The analytical procedures were performed using the R software package (https://www.r-project.org/). An initial one-way analysis of variance (ANOVA) was carried out to assess overall variations across the groups of interest. Subsequently, to delve into specific inter-group differences, a Tukey post hoc test was employed. This comprehensive statistical analysis was conducted with GraphPad Prism version 10.0.0 for Windows, GraphPad Software, Boston, MA, USA, www.graphpad.com.

## 3. Results

### 3.1. Gross Anatomy

**Figure 1:**
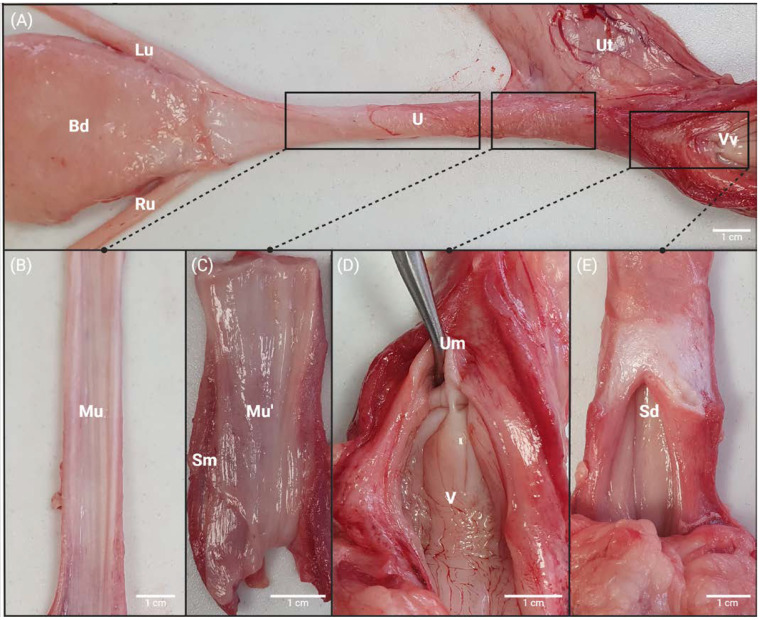
Macroscopic anatomy of the sow urethrovesical unit. (**A**) Dorsal aspect of the lower urinary tract; (**B**) longitudinal section of the Pu and MPu region; (**C**) longitudinal section of the MDu region; (**D**,**E**) longitudinal section of the vaginal vestibulum. White marks: Bd—bladder, Ru—right Ureter, Lu—left ureter, U—urethra, Ut—uterus, Vv—vagina. Mu—mucosal tissue of the urethra, Mu’—mucosal tissue of the urethra in MD region, Sm—striated muscle, Um—external urethral orifice (urinary meatus), V—vaginal mucosa, Sd—suburethral diverticulum. Size bar: 1 cm.

The female pig urethra is encased in connective tissue and exhibits muscular attachments to the pelvic floor. Following the removal of surrounding connective tissue, its length measures approximately 11 cm (Figure 1A). The proximal, mid-proximal and mid-distal urethra share similar macroscopic characteristics, varying primarily in height: 1.5 cm for the proximal portion, 0.89 cm for the middle portion, and 1.2 cm for the distal portion. Upon observing a longitudinal section of the distal urethra, a distinct region of approximately 2 cm is discernible. This area appears notably redder and exhibits increased thickness when compared to the other segments of the urethra (Figure 1B,C).

In its proximal portion, the urethra is closely associated with the bladder, while it terminates towards the distal tract at the external urethral orifice. Notably, this termination is characterized by the presence of the suburethral diverticulum (Figure 1E). Detailed measurements of the various urethral segments are consolidated in Table 1.

Based on the measurements provided Table 1, it was determined that 27.3% of the entire urethral length comprised the proximal and distal portions, whereas 45.4% was attributed to the middle portion, further divided evenly between the mid-proximal and mid-distal segments.

### 3.2. Histological and Morphometric Analysis

**Figure 2:**
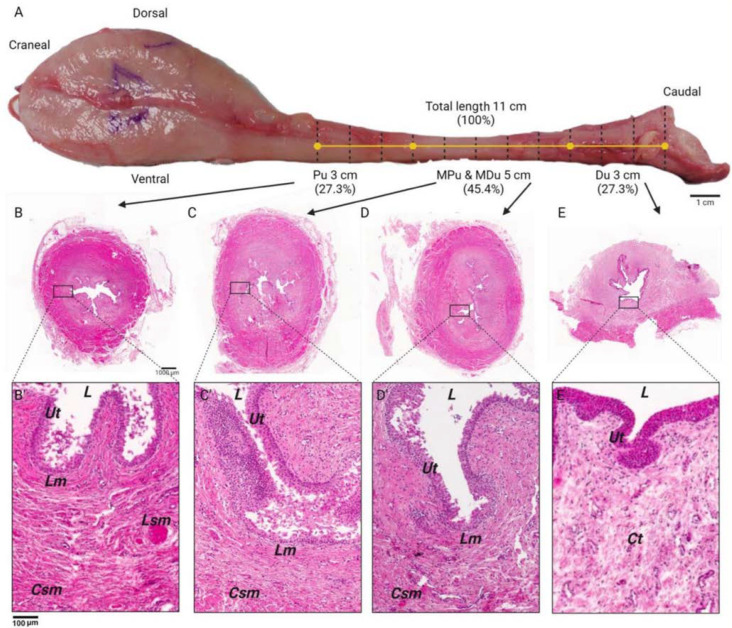
Microscopic characterization of the cellular territory of the sow’s urethra. (**A**) Proportion of urethral segments. Photomicrographs showing the structure of the portions of the female pig urethra in corresponding cross-sections stained with Hematoxylin-eosin and viewed with light microscopy; (**B**) proximal urethra; (**C**) mid-proximal urethra; (**D**) mid-distal urethra; (**E**) distal urethra. (**B’**–**E’**) Areas identified in the histological sections of the female pig urethra. L—lumen, Ut—urothelium, Lm—lamina propia, Lsm—longitudinal smooth muscle, Csm—circular smooth muscle, Ct—connective tissue. Size bar. 1 cm (panel (**A**)), 100 mm (panels (**B**–**E**)).

Microscopic examination revealed distinctive features within the urethral lumen across different portions. The proximal and middle segments displayed a pleomorphic central arrangement, transitioning to an adapted star-like configuration in the distal portion. Notably, the lining epithelium varied among these sections: the proximal urethra exhibited transitional epithelium, the distal segment featured nonkeratinized stratified squamous epithelium, and the middle portion was lined with pseudostratified epithelium (Figure 2).

Numerous layers of epithelial cells were evident at the proximal urethral outset, transitioning to fewer cell layers towards the section’s terminus. Across all urethral portions, acinar glands were sparsely present, accompanied by an abundance of blood vessels.

**Figure 3:**
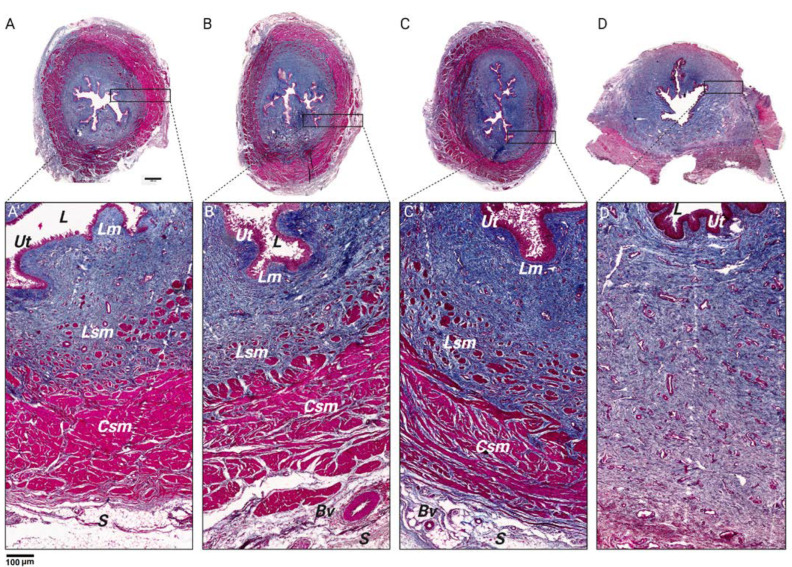
Microscopic characterization of the muscular territory of the sow’s urethra. Photomicrographs showing the structure of the portions of the female pig urethra in corresponding cross-sections stained with Masson trichrome and captured with light microscopy. (**A**) Proximal urethra; (**B**) mid-proximal urethra; (**C**) mid-distal urethra; (**D**) distal urethra. L—lumen, Ut—urothelium, Lm— lamina propia, Lsm—longitudinal smooth muscle, Csm—circular smooth muscle, S—serous, Bv—blood vessel. Size bar: 1000 mm (panels (**A**–**D**)), 100 mm (panels (**A’**–**D’**)).

In each study segment, except for the distal region, a substantial number of muscle fibers were evident, arranged both longitudinally and transversely. Particularly noteworthy was the complete encirclement of the urethral diameter by these fibers in the proximal and middle regions (Figure 3).

**Figure 4:**
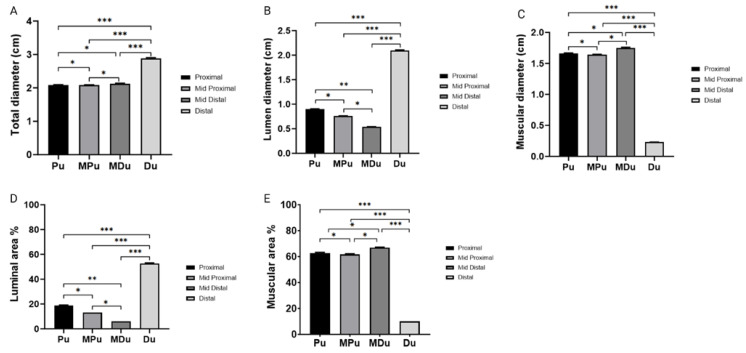
Tissue composition among the regions of the sow’s urethra. (**A**) Total urethral diameter; (**B**) urethral lumen space; (**C**) urethral muscle space; (**D**) total urethral area occupied by the lumen; and (**E**) total urethral area occupied by the muscle. The asterisks above the bars in the graphs indicate significant differences in the histomorphometric analysis (* *p* < 0.05; ** *p* < 0.01; *** *p* < 0.001; ANOVA with Tukey post hoc test).

The morphometric parameters provide critical findings concerning the luminal and muscular diameter of the female pig urethra across various segments, including the proximal, mid-proximal, mid-distal, and distal regions (Figure 4).

Our analysis provides comprehensive insights into the variations in the total diameter of the sow’s urethra, revealing significant differences across distinct anatomical regions. The proximal region exhibited a 27.8% reduction in total diameter compared to the distal region, while the mid-proximal region showed a 27.1% decrease, and the mid-distal region manifested a more substantial 35.4% reduction. Both the mid-distal and mid-proximal segments demonstrated significantly smaller total diameter measurements than the distal region (*p* < 0.001). Additionally, the proximal region displayed total diameter measurements significantly lower than those of the distal region (*p* < 0.001).

Significant luminal diameter variations were observed among the urethral segments. The mid-proximal region showed a reduction of approximately 15.6% compared to the proximal urethra. In contrast, the mid-distal region demonstrated a more substantial decrease of about 41.1%. Remarkably, the distal urethra exhibited an increase of 132.2% in luminal diameter compared to the proximal urethra. In the mid-distal segment, the luminal diameter was significantly smaller than that of the distal segment (*p* < 0.001). Similarly, both the mid-proximal and proximal segments displayed significantly reduced luminal diameters compared to the distal segment (*p* < 0.001).

The analysis also revealed significant variations in muscular diameter across different anatomical regions. The mid-proximal region exhibited a 1.8% reduction compared to the proximal region. In contrast, the mid-distal region demonstrated a 4.8% increase. The distal region displayed an 86.7% reduction in muscle diameter compared to the proximal region. Both the mid-distal and mid-proximal segments show substantially larger muscular diameters than the distal region (*p* < 0.001). Furthermore, the proximal segment exhibited muscular diameter measurements significantly greater than those of the distal region (*p* < 0.001). Moreover, significant differences in luminal area variations exist within the female pig urethra among anatomical regions. The mid-proximal region exhibited a decrease of approximately 32.0% compared to the proximal urethra. In contrast, the mid-distal region demonstrated a more substantial decrease of about 68.4%. The distal region manifested a significant increase of approximately 173.7% in luminal area compared to the proximal urethra. Both the mid-distal and mid-proximal segments displayed luminal area measurements significantly smaller than those of the distal region (*p* < 0.001). Additionally, the proximal region exhibited luminal area measurements significantly smaller than those of the distal region (*p* < 0.001).

Finally, the results unveil significant differences in muscular area measurements among these regions. The mid-proximal region exhibited a decrease of approximately 3.2% compared to the proximal urethra. In contrast, the mid-distal region demonstrated a slight increase of about 6.6%. Remarkably, the distal region manifested a substantial decrease of approximately 99.8% in luminal area compared to the proximal urethra. The mid-distal and mid-proximal regions exhibited muscular area measurements significantly larger than those of the distal region (*p* < 0.001). Moreover, the proximal region displayed muscular area measurements that were considerably larger than those of the distal region (*p* < 0.001).

### 3.3. Urodynamic Studies

**Figure 5:**
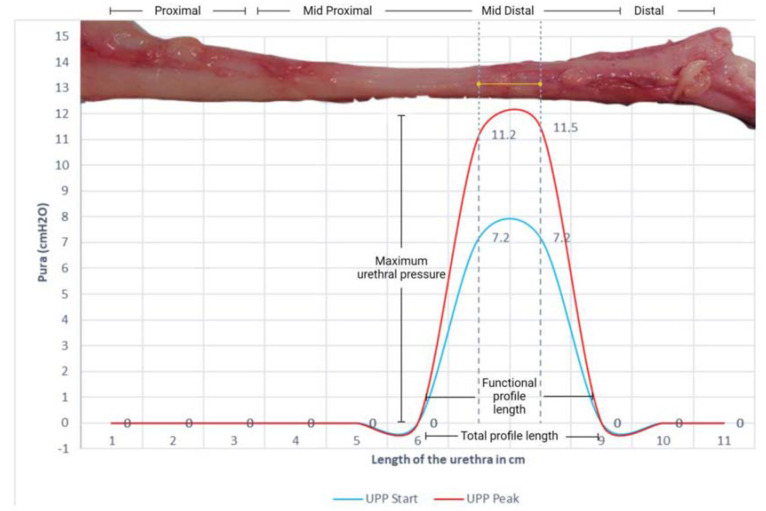
Urinary profilometries conducted in female pigs reveal that the point of maximum urethral pressure occurs in the mid-distal segment along the functional profile length.

The urethral pressure profile (UPP) was assessed to gain insights into the urethral pressure (Pura) characteristics. The start UPP, which represents the baseline urethral pressure at rest, was measured and found to be 7.2 cmH2O. Furthermore, the UPP peak, signifying the maximum urethral pressure during bladder activity, was determined. The UPP peak was recorded at 11.5 cmH2O, reflecting the urethral pressure at its highest point during the procedure (Figure 5).

## 4. Discussion

Over the last two decades, pigs have become increasingly favored as biomedical models for a wide array of studies, encompassing surgical training, research endeavors, and the advancement of novel techniques [24]. Their utilization spans various medical procedures, such as general surgery, laparoscopy, endoscopy, transplants, traumatic interventions, and device implantations, among others. The choice of large animal models, particularly pigs, offers distinct advantages due to their body and organ size, which closely resemble those of humans. These anatomical similarities enable researchers to employ tools and techniques analogous to those used in clinical settings, thereby augmenting the relevance and applicability of the research findings [25].

It is possible to induce urinary incontinence in animal models through various methodologies, depending on the specific mechanism being studied. Significant urethral sphincter deficiency can be induced in female pigs using a combination of urethral dilatation and distal electrocautery [15]. This deficiency can be effectively measured through standard and high-definition urethral pressure profilometry, and has been shown to persist for at least 21 days post-induction, correlating with visible changes in the tissue structure of the distal urethra. In alignment with our observations, the female pig urethra has been previously described with a straight anatomical configuration, concluding distally into the vagina on the ventral wall [24]. However, our anatomical examination uncovered a variance in the reported length of the urethra compared to earlier data. This difference in measurement may be ascribed to variations in the pig breed employed for our study. Notably, the female urethra is recognized for its considerable variability, with reported lengths ranging from 1.9 to 4.5 cm [26]. Upon comparing our findings with those of the sow’s urethra, a noteworthy distinction becomes evident. The variation in length presents opportunities to model uroendoscopic and surgical approaches more comprehensively and conveniently, especially in the context of applications for women.

Our findings closely correspond with the outcomes of previous research investigating pig urethral histology. The persistent presence of transitional epithelium in the bladder neck and proximal urethra of female pigs aligns with observations from earlier studies on porcine urothelial morphology. The identification of nonkeratinized stratified squamous epithelium in the distal segments, coupled with transitional epithelium in the intermediate region, underscores a notable histological similarity between pig and human urethras [27]. These consistent findings not only validate the fidelity of the pig model in representing anatomical features but also underscore its histological congruence. These similarities suggest adaptive mechanisms in response to varying urinary volumes and protection against mechanical stress, indicative of urothelial plasticity required to maintain urinary function under dynamic physiological conditions.

Earlier investigations into the morphological aspects of urethral muscles have delineated discernible layers comprising longitudinal smooth muscle, circular smooth muscle, and striated muscle [28]. Additionally, both urethral structures shared substantial layers of smooth muscle fibers, oriented both longitudinally and transversely [29,30]. This muscular arrangement indicates the role of smooth muscle in facilitating peristaltic contractions during urine expulsion, a fundamental function conserved across species. A notable difference in muscle distribution was observed between the female pig and human urethras. In humans, muscle fibers formed a semi-enveloping pattern resembling an omega shape [31]. In contrast, in the female pig urethra, muscle fibers covered the entire urethral diameter, except for the distal region. This divergence raises questions regarding potential functional implications, such as variations in urethral support and contractility, which may impact urinary continence and control mechanisms, akin to those observed in human females [32,33].

Finally, our comprehensive urodynamic studies have provided valuable insights into the intricate characteristics of urethral pressure dynamics. However, careful consideration is essential in interpreting these findings, given the observed disparities in urinary profilometry results compared to previously reported pig and human values [18,34]. Regarding the election of female pigs instead of male pigs, there is limited literature comparing the male and female urethra in pigs; however, one study specifically investigates how lumen roughness and urethral length influence urinary flow speed, utilizing tomographic analysis to differentiate between sexes. This study highlights key structural differences: female pigs have nozzle-shaped urethras that merge with a segment of the reproductive tract, whereas male pigs exhibit pipe-shaped urethras [35]. Regarding our study limitations, we have a small number of animals (six sows), so our results are rather preliminary and could not reflect a general picture; a larger cohort is needed for future efforts and generalization of results. The variations noted in both the baseline urethral pressure at rest and the maximum urethral pressure raise questions about potential factors influencing these discrepancies. It is crucial to acknowledge that the anesthetic procedures employed during our studies may have contributed to muscle relaxation in the urethra, impacting the recorded pressure profiles. Therefore, it is imperative to recognize that our findings may reflect an altered physiological state due to anesthesia rather than inherent differences in urethral function between the female pig and human. Likewise, advanced imaging or biomechanical modeling could provide deeper insight in future studies. Further investigation is warranted to elucidate the extent of anesthesia-induced effects on urethral pressure dynamics and to refine our understanding of the female pig urethra as a model for urological research.

## 5. Conclusions

In conclusion, our study underscores the utility of the female pig as a translational model for urological research, offering anatomical and physiological similarities to humans that facilitate the investigation of urinary incontinence pathogenesis and future therapeutic interventions.

## Figures and Tables

**Table 1:** Measurements of the urethra of the female pig.

Variable	Min–Max	Mean	SD
Total length (cm)	10.7–11.1	10.9	0.2
Proximal length (cm)	0.9–1.0	0.9	0.1
Mid-proximal length (cm)	3.1–3.3	3.2	0.07
Mid-distal length (cm)	5.0–5.2	5.1	0.1
Distal length (cm)	2.3–2.8	2.7	0.2

## Data Availability

All relevant data are provided upon request.
